# Diagnostic accuracy of automated hematology analyzer abnormal flags for detecting hematological malignancies: A systematic review and meta-analysis

**DOI:** 10.1371/journal.pone.0354619

**Published:** 2026-07-31

**Authors:** Zewudu Mulatie, Bruktawit Eshetu, Afewerk Habtamu, Sisay Desale, Saleamlak Sebsibe, Yonas Erkihun, Yeshimebet Kassa, Tesfaye Gessese, Mihreteab Alebachew, Mikiyas Shimeles, Mahider Shimelis Feyisa, Dereje Mengesha Berta

**Affiliations:** 1 Department of Medical Laboratory Sciences, College of Medicine and Health Sciences, Wollo University, Dessie, Ethiopia; 2 School of Medical Laboratory Sciences, Asrat Weldeyes Health Science Campus, Debre Berhan University, Debre Berhan, Ethiopia; 3 Department Hematology and immunohematology, School of Biomedical and Laboratory Science, College of Medicine and Health Science, University of Gondar, Gondar, Ethiopia; University of Diyala College of Medicine, IRAQ

## Abstract

**Background:**

Hematological malignancies including leukemia, lymphoma, and myelodysplastic syndromes, are characterized by clonal proliferation of abnormal blood or bone marrow cells. Early and accurate detection is essential for improving treatment outcomes and survival. Automated hematology analyzers generate abnormal flags that may indicate underlying hematologic malignancies; however, their overall diagnostic accuracy has not been comprehensively evaluated. This systematic review and meta-analysis aimed to assess the diagnostic performance of abnormal flags for detecting hematological malignancies.

**Methods:**

A systematic search of PubMed, PubMed Central, Scopus, ScienceDirect, and Google Scholar was conducted to identify relevant diagnostic accuracy studies. Methodological quality was evaluated using the Quality Assessment of Diagnostic Accuracy Studies-2(QUADAS-2) tool. Pooled sensitivity, specificity, positive likelihood ratio, negative likelihood ratio, and diagnostic odds ratio were calculated using a bivariate random-effects model in Stata version 17.0. Heterogeneity was assessed using the I^2^ statistic, and subgroup and meta-regression analyses were performed to explore potential sources of variability.

**Results:**

Twenty-eight studies met the inclusion criteria. The pooled sensitivity and specificity of abnormal hematology analyzer flags for detecting hematological malignancies were 91% (95% CI: 87%–94%) and 89% (95% CI: 84%–92%), respectively, indicating good diagnostic accuracy. Significant heterogeneity was observed across studies (I^2^ > 50%). Meta-regression analysis identified the type of abnormal flag as a significant source of heterogeneity in sensitivity (p < 0.001), whereas both the type of abnormal flag and the analyzer platform significantly influenced specificity.

**Conclusion:**

Automated hematology analyzer abnormal flags showed promising diagnostic performance. However, substantial heterogeneity and differences in analyzer platforms, flag types, and reference standards reduce the certainty and generalizability of pooled estimates. Nevertheless, these findings support the use of abnormal hematology analyzer flags as an effective initial screening tool in routine laboratory practice, particularly in resource-limited settings where rapid and cost-effective diagnostic support is essential. **Systematic review registration** PROSPERO (CRD42024601908**).**

## Introduction

Hematological malignancies, including leukemia, lymphoma, and myelodysplastic syndromes (MDS), comprise a diverse group of disorders characterized by the clonal proliferation of abnormal blood or bone marrow cells [1,2]. Early and accurate diagnosis is essential for timely treatment and improved patient outcomes. The complete blood count (CBC)is often the first diagnostic test performed when a hematologic disorder is suspected. Automated hematology analyzers, including Sysmex and Beckman Coulter platforms are widely used in modern laboratories to perform CBCs, providing rapid, high-throughput analysis while reducing operator-dependent variability [3–5].

Several clinical and laboratory findings may raise suspicion of an underlying hematological malignancy, including impaired bone marrow function, splenomegaly, lymphadenopathy, tissue destruction, and increased metabolic activity. Although these findings may represent early manifestations of hematological malignancies, they should always be interpreted in conjunction with the patient’s overall clinical presentation [6,7]. In recent years, hematological malignancies have increasingly been detected incidentally during routine health examinations, particularly among asymptomatic individuals with chronic diseases. For example, a markedly elevated white blood cell (WBC) count identified during routine CBC testing may be the first indication of previously undiagnosed chronic leukemia [8].

Both automated cell counters and peripheral blood film examination are essential for diagnosing a variety of hematological disorders. Automated hematology analyzers are particularly valuable for detecting abnormal blood cell populations that may indicate hematological malignancies or other hematologic abnormalities [9]. By distinguishing normal from abnormal scattergram patterns, these analyzers can identify conditions such as leukocytosis, leukopenia, and leukemia. They also generate scattergrams that provide valuable diagnostic information while substantially reducing turnaround time, particularly in high-volume laboratory settings [10].

Modern analyzers combine conventional hematological parameters with advanced detection technologies, including optical scatter analysis, fluorescence flow cytometry, and electrical impedance measurements. Using sophisticated algorithms, they generate abnormal flags when morphological abnormalities are suspected. These flags, including blast flags, abnormal lymphocyte flags, immature granulocyte flags, and abnormal monocytosis flags, serve as early warning indicators that prompt further diagnostic investigations, such as flow cytometry, bone marrow examination, or peripheral blood smear review [11,12].

Abnormal flagging offers several advantages over conventional diagnostic workflows. It enables real-time identification of potentially malignant samples, facilitating early screening and prioritization of high-risk patients. It also improves laboratory efficiency by reducing unnecessary peripheral blood smear reviews and focusing resources on samples requiring further investigation. Furthermore, abnormal flags can be integrated with advanced decision-support parameters, such as Cell Population Data (CPD) and Monocytosis Workflow Optimization (MWO), to enhance predictive performance. These approaches improve cost-effectiveness in high-volume laboratories while promoting standardized and reproducible results by reducing the inter-observer variability associated with manual microscopy [13,14].

Despite their clinical importance, currently available diagnostic methods have important limitations as first-line screening tools. Although manual peripheral smear examination is still the gold standard for morphological evaluation, it is it is labor-intensive, time-consuming, subjective, and unsuitable for large-scale, high-throughput screening [15]. Flow cytometry provides highly accurate immunophenotypic characterization but is expensive, technically demanding, and impractical for routine initial screening because of its cost and longer turnaround time [16]. Similarly, cytogenetic and molecular diagnostic techniques are indispensable for definitive diagnosis and prognostic assessment but remain costly, technically complex, and unsuitable for rapid screening of large populations. In addition, CBC parameters alone may fail to detect early-stage hematological malignancies in the absence of marked quantitative abnormalities [17,18].

Recent advances in hematology analyzer technology have substantially improved the sensitivity for detecting abnormal circulating blood cells. However, maintaining an appropriate balance between diagnostic sensitivity and unnecessary follow-up investigations remains a major challenge [12]. Consequently, modern automated hematology analyzers provide a rapid, objective, and scalable screening approach that bridges routine hematology testing and definitive diagnostic procedures through abnormal flagging. Although their diagnostic performance may vary according to analyzer platform, flagging algorithm, patient population, and disease spectrum, these systems have considerable potential as initial screening tools. Therefore, a systematic review and meta-analysis is needed to comprehensively evaluate the diagnostic accuracy of abnormal hematology analyzer flags across different analyzer platforms and flag types.

## Methods

### Reporting guideline and protocol registration

This systematic review and meta-analysis was conducted and reported in accordance with the Preferred Reporting Items for Systematic Reviews and Meta-Analyses (PRISMA) statement (S1 File). The study protocol was prospectively registered in the International Prospective Register of Systematic Reviews (PROSPERO; registration number CRD42024601908).

### Search strategy

A comprehensive literature search was conducted between September and October 30, 2025, using PubMed, Embase, the Cochrane Library, ScienceDirect, Scopus, and Google Scholar. The search strategy included combinations of the following keywords: “hematology analyzer,” “flag,” “abnormal flagging,” “blast flag,” “WBC flag,” “alarm flag,” “white blood cell disorders,” “leukemia,” “lymphoma,” “myelodysplasia,” “diagnostic accuracy,” “predictive value,” “performance,” and “hematologic malignancy.” Appropriate Boolean operators (AND and OR) were used to combine search terms. In addition, the reference lists of all eligible articles were manually screened to identify potentially relevant studies. The complete search strategies for each database are provided in (S2 File).

### Inclusion and exclusion criteria

Eligible studies were required to meet predefined inclusion criteria. Specifically, observational studies (cross-sectional, case–control, and cohort studies) evaluating the diagnostic accuracy of abnormal hematology analyzer flags for detecting hematological malignancies were included. Studies were required to analyze whole-blood samples and report sufficient diagnostic accuracy data (e.g., sensitivity, specificity, or data enabling calculation of true positives [TP], false positives [FP], true negatives [TN], and false negatives [FN]). When a single publication reported diagnostic performance for multiple hematology analyzer platforms using the same study population, each analyzer platform was treated as a separate index-test dataset because it represented a distinct diagnostic technology.

Studies were excluded if they were conference abstracts, letters, editorials, commentaries, narrative reviews, systematic reviews, meta-analyses, animal studies, or did not provide sufficient data to construct 2 × 2 contingency tables or estimate diagnostic accuracy measures.

### Study selection, data extraction, and quality assessment

All retrieved records were imported into EndNote version 20, and duplicate records were removed. Three reviewers (ZM, MSF, and DMB) independently screened titles, abstracts, and full-text articles for eligibility. Disagreements were resolved through discussion or consultation with a fourth reviewer (BE). Four reviewers (AHA, SD, SS, and MS) independently extracted data using a structured Microsoft Excel data extraction form developed by consensus. The extracted information included study characteristics (first author, country, hematology analyzer model, abnormal flag type, reference standard, and sample size) and diagnostic accuracy measures (sensitivity, specificity, area under the curve [AUC], TP, FP, TN, and FN). All extracted data were cross-checked for accuracy and consistency. Any discrepancies were resolved through consensus, and when necessary, a senior reviewer (ZM) was consulted. The methodological quality of the included studies was evaluated using the Quality Assessment of Diagnostic Accuracy Studies-2 (QUADAS-2) tool. This tool assesses four domains: patient selection, index test, reference standard, and flow and timing. Quality assessment was performed independently by three reviewers (YE, YK, and TG), and disagreements were resolved through discussion or consultation with another reviewer (MA).

### Data analysis

Data were analyzed using Stata version 17.0 (StataCorp, USA). A bivariate random-effects diagnostic accuracy model was used to estimate pooled sensitivity, specificity, positive likelihood ratio (PLR), negative likelihood ratio (NLR), diagnostic odds ratio (DOR), and their corresponding 95% confidence intervals (CIs)based on extracted TP, FP, TN, and FN values. Forest plots were generated to summarize individual study estimates, and summary receiver operating characteristic (SROC) curves were constructed to estimate the area under the curve (AUC) as an overall measure of diagnostic performance.

Because the included studies evaluated different hematology analyzer-generated abnormal flags across various hematological malignancies, pooled analyses were performed to estimate overall screening performance. Subgroup analyses according to abnormal flag type and analyzer platform were conducted to explore potential sources of clinical heterogeneity.

Sensitivity analyses were performed to evaluate the robustness of the pooled estimates. Publication bias was assessed using Deeks’ funnel plot asymmetry test. Clinical utility was evaluated using Fagan nomograms and likelihood ratio scattergrams. Statistical heterogeneity was assessed using Cochran’s Q test and the I^2^ statistic. Heterogeneity was considered statistically significant when p < 0.05 for Cochran’s Q test and I² > 50%. A two-sided p value < 0.05 was considered statistically significant.

### Ethical approval and consent to participate

Ethical approval and consent to participate were not required because this was a systematic review and meta-analysis that did not involve human or animal experiments.

## Result

### Description of included studies

**Fig 1:**
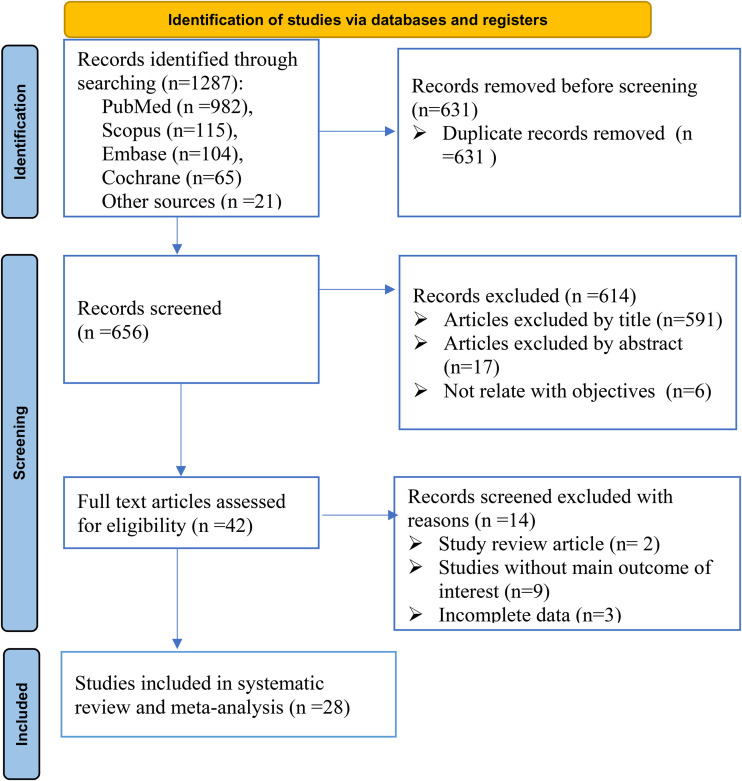
Flow diagram of the included studies for the systematic review and meta-analysis.

A total of 1,287 records were identified through database searching. After removal of 631 duplicate records, 656 unique records remained for title and abstract screening. Of these, 614 records were excluded because they did not meet the eligibility criteria. Forty-two full-text articles were assessed for eligibility, of which 14 were excluded for the reasons presented in the PRISMA flow diagram. Ultimately, 28 articles comprising 57 individual diagnostic accuracy datasets were included in the final systematic review and meta-analysis (Fig 1).

### Characteristics of the included studies

A total of 28 articles comprising 57 individual diagnostic accuracy datasets were included in this review. Of these, 26 datasets originated from Asia, 20 from Europe, 8 from Africa, and 3 from the Americas. Microscopic examination served as the reference standard in most studies. The included studies evaluated abnormal flags generated by several hematology analyzer platforms. Sysmex analyzers were used in 37 datasets, whereas Beckman Coulter analyzers were used in six datasets. Twenty-five studies evaluated blast flags alone, while 20 studies assessed combinations of abnormal flags for the detection of hematological malignancies (Table 1).

**Table 1:** Characteristics of the included studies.

S.no.	Authors	Dataset	Year	County	Analyzer model	Flag type	Reference Standard	Sample Size	Sensitivity	Specificity
1	Tohyama et al. [19]	A	2005	Japan	Sysmex XE 2100	Blast and Multi-Scorin gProgram	Microscope	321	83	90
		B	2005	Japan	Sysmex XE 2100	Blast and multi-Scoring Program	Microscope	171	46	88.3
2	Shelat et al. [20]	A	2008	USA	ADVIA 2120	Blast	Microscope	390	100	49.3
		B	2008	USA	LH750	Blast	Microscope	390	62.1	85.6
3	Briggs et al. [21]	A	2012	UK	XE-2100	Blast cell flag	peripheral blood smear	390	100	82
		B	2012	UK	Sysmex XN series	Blast cell flag	peripheral blood smear	390	95	99.7
4	Meintker et al. [22]	A	2013	Germany	Sapphire	Blast	peripheral blood smear	202	65	86
		B	2013	Germany	Advia 120	Blast	peripheral blood smear	202	71	67
		C	2013	Germany	Sysmex XE-2100	Blast	peripheral blood smear	202	94	80
		D	2013	Germany	Beckman coulter DxH 800	Blast	peripheral blood smear	202	82	80
5	Bruegel et al. [23]	A	2014	Germany	Sapphire	Blasts and/or variant lymphocytes and/or immature granulocytes	Microscope	349	68	88
		B	2014	Germany	Beckman coulter DxH 800	Blasts and/or variant lymphocytes and/or immature granulocytes	Microscope	349	68	88
		C	2014	Germany	Advia 2120i	Blasts and/or variant lymphocytes and/or immature granulocytes	Microscope	349	64	89
		D	2014	Germany	Sysmex XE-5000	Blasts and/or variant lymphocytes and/or immature granulocytes	Microscope	349	85	88
		E	2014	Germany	XN-2000	Blasts and/or variant lymphocytes and/or immature granulocytes	Microscope	349	98	78
6	Becker et al. [24]		2015	France	Sysmex XN 1000	blast, abnormal lymphocyte	Microscope	161		
7	Schuff-Werner et al. [25]		2016	Germany	Sysmex XN	WPC channel	standard morphology, immune pheno typing and clinical diagnosis	253	93.7	92.8
8	Furundarena et al. [26]	A	2016	Spain	Sysmex XE‑5000	blast & abnormal lymphocyte flags	CellaVision + Hematologist	292	59.3	88.3
		B	2016	Spain	Sysmex XN-2000	blast & abnormal lymphocyte flags	CellaVision + Hematologist	292	70.9	91.3
9	Schillinger et al. [27]		2017	France	Sysmex XN TM	n/m ratio, (Ne-WX) and monocyte absolute value	PBS microscopy	696	96.7	97.8
10	Genc et al. [28]	A	2017	Turkey	XN 3000	Blast	Microscope	102	72.2	67.8
		B	2017	Turkey	XN 3000	Blast	Microscope	102	55.5	89.2
11	Schapkaitz et al. [29]	A	2018	South Africa	Sysmex XN-9000	Immature granuloctyes	peripheral blood smear	211	81	82
		B	2018	South Africa	Sysmex XN-9000	Blasts/Abnormal lymphocytes	peripheral blood smear	218	100	73
		C	2018	South Africa	Sysmex XN-9000	Atypical lymphocytes	peripheral blood smear	209	74	73
		D	2018	South Africa	Sysmex XN-9000	Blasts (WPC flag)	peripheral blood smear	196	100	90
		E	2018	South Africa	Sysmex XN-9000	Abnormal lymphocyte (WPC flag)	peripheral blood smear	182	100	94
12	Aidoudi et al. [30]		2019	France	ADVIA 2120/ 2120i	blast flag	Blood smear pathology	115603	89.4	98.97
13	Petrone et al. [31]		2019	USA, tertiary care	Sysmex XN‑10	combined “blasts?/abn lymph?” + IG flags analysis	CellaVision image analysis	2239	100	50.2
14	Eilertsen et al. [32]	A	2019	Norway	Sysmex XE	blast flags	Flow cytometry	240	40.9	81.6
		B	2019	Norway	Sysmex XN	Blasts/Abn Lympho	Flow cytometry	240	89.0	18.4
15	Blomme et al. [33]		2020	Belgium	Sysmex XN-9100 (WPC)	Blasts and Abnormal Lymphocytes	Manual smear (DI-60)	420	99	29
16	Sejrup et al. [34]	A	2020	Denmark	Sysmex XN-20 (WPC)	Blasts	Manual WBCC (CellaVision)	117	40	82
		B	2020	Denmark	Sysmex XN-20 (WPC)	Blasts and all WDF and WPC DIFF	Manual WBCC (CellaVision)	117	88	82
17	Joshi et al. [35]	A	2020	India	Mindray BC-6800	Blasts	Microscope	500	75.81	98.4
		B	2020	India	Mindray BC-6800	abnormal lymphocyte flags	Microscope	500	100	94.5
		C	2020	India	Mindray BC-6800	Atypical lymphocyte	Microscope	500	100	97.5
		D	2020	India	Mindray BC-6800	Imature granulocyte	Microscope	500	90.5	99.48
18	Kocaturk et al. [36]	A	2020	Turkey	Sysmex XN series	Monocytosis	Microscope	155	90.5	76.9
		B	2020	Turkey	Sysmex XN series	Monocytosis with abnormal lymphocyte/ blast”	Microscope	155	100	68.7
19	Gupta et al. [37]		2020	India	Sysmex XT-2000i	Blast	Microscope	113	73.33	96.94
20	Permatasari et al. [38]		2021	Indonesia	Sysmex XN 1000	WPC Channel Scattergram	blood and BM aspiration and immunophenotype	86	85.7	91.89
21	Querol et al. [39]	A	2021	Spain	Sysmex XN-20	Blast	Microscope	913	100	84.9
		B	2021	Spain	DxH900	Blast	Microscope	913	87.5	98.8
22	Eldanasoury et al. [40]		2023	Egypt	Sysmex XN-1000	Blast/Abnormal lymphocytes, Atypical lymphocyes, Left Shift, and Immature granulocytes	Manual smear review	400	100	79.8
23	Ramiah et al. [41]	A	2023	South Africa	Sysmex XN‑3000	Blast	PBS microscopy	250	96.3	84.9
		B	2023	South Africa	Sysmex XN‑3000	abnormal lymphocyte flags	PBS microscopy	250	90	96.2
24	Kim et al. [42]		2023	Korea	Beckman Coulter DxH 900	WBC-related flags+ CPD rule	Microscope	239	97.5	71.9
25	Fujimaki et al. [43]	A	2024	Germany	Sysmex Xn	WPC Abnormal flags (Blasts? or Abn Lympho?)	Microscope	926	97	88.3
		B	2024	Germany	Sysmex XR	WPC Abnormal flags (Blasts or Abn Lympho?)	Microscope	926	96	86.8
26	Tailor et al. [44]		2024	India	DXH 800	WBC scatterplot patterns	BM aspiration and biopsy and immunophenotype	272	90	98.4
27	Schoorl et al. [45]	A	2016	Netherlands	Sysmex XN 2000	Blasts	Microscope	2011	100	99.7
		B	2016	Netherlands	Sysmex XN 2000	Atypical lymph	Microscope	2011	78.9	100
28	Graaf et al. [46]	A	2025	Netherlands	Yumizen H2500	Blast	microscope and clinical	377	87.1	42.5
		B	2025	Netherlands	Yumizen H2500	blast, atypical lymphocyte, immature granulocyte	microscope and clinical	377	93.3	55.2
		C	2025	Netherlands	Yumizen H2500	atypical lymphocyte	microscope and clinical	377	77.5	72.4
		D	2025	Netherlands	Yumizen H2500	immature granulocyte	microscope and clinical	377	84.7	78.7

**NB**: Multiple datasets from the same publication represent separate evaluations of different analyzer platforms or abnormal flag categories within the same study population. Dataset (e.g., A–D) indicate multiple evaluations originating from the same publication.

### Data quality assessment

**Fig 2:**
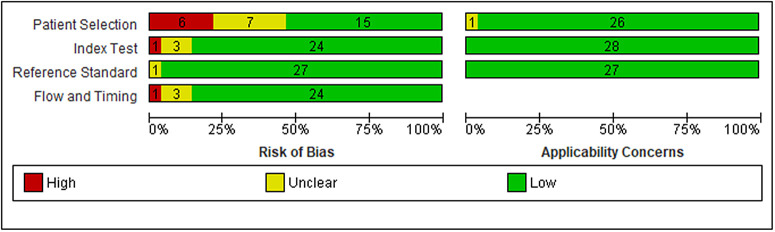
Risk of bias graph of studies included in the meta-analysis.

Of the 28 diagnostic studies, 15 were deemed to have a low risk of bias in the patient selection area, whereas six showed a high risk. In terms of applicability issues, none of the investigations indicated that patient selection carried a substantial risk. One study in the flow and timing area was deemed to have a significant risk of bias (Fig 2).

### Diagnostic accuracy of abnormal flags

The forest plots illustrating the pooled diagnostic performance of abnormal hematology analyzer flags for detecting hematological malignancies are presented below. The combined estimates showed a sensitivity of 0.91 (95% CI: 0.87–0.94) and a specificity of 0.89 (95% CI: 0.84–0.92). The pooled positive likelihood ratio (PLR) was 8.11 (95% CI: 5.68–11.58), while the negative likelihood ratio (NLR) was 0.10 (95% CI: 0.07–0.15). The diagnostic odds ratio (DOR) was 80.28 (95% CI: 45.90–140.42).

**Fig 3:**
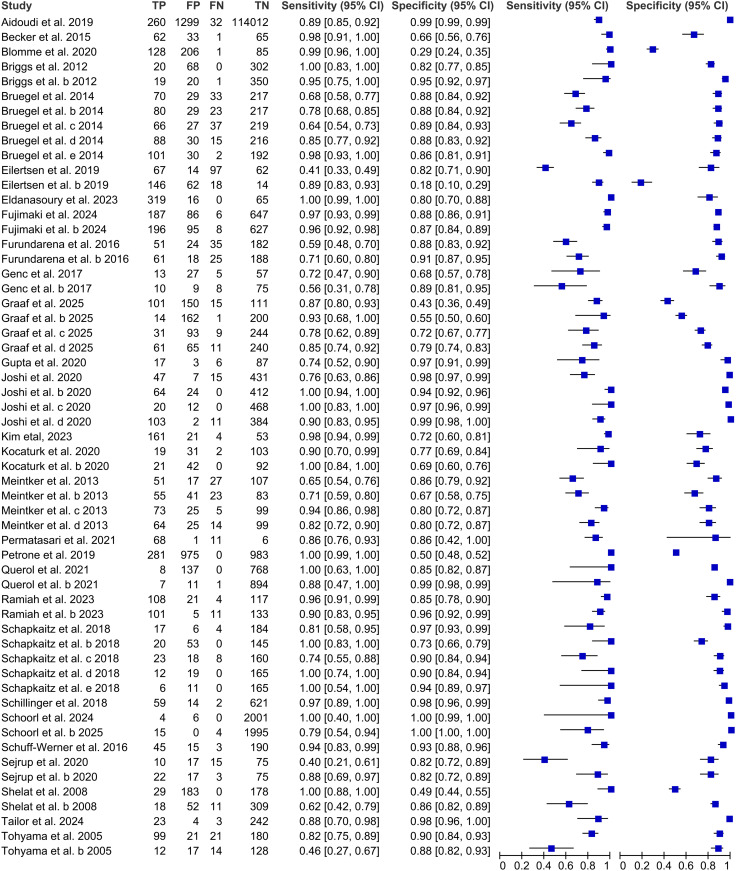
Forest plot of abnormal flags for hematological malignancy diagnosis.

**Fig 4:**
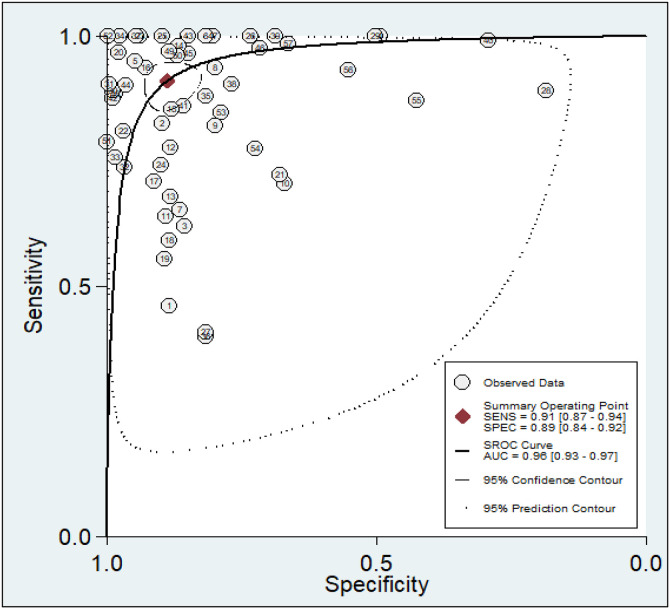
SROC, 95% confidence contour and 95% prediction contour.

Given the substantial heterogeneity observed across studies for both sensitivity (I^2^ = 94.48%) and specificity (I^2^ = 99.73%), a bivariate random-effects model was applied to derive the pooled diagnostic estimates (Fig 3) (Table 2). The summary receiver operating characteristic (SROC) curve demonstrated an area under the curve (AUC) of 0.96 (95% CI: 0.93–0.97), indicating excellent overall diagnostic accuracy. This high AUC value highlights the strong diagnostic capability of abnormal flags in identifying hematological malignancies (Fig 4).

**Table 2:** Summary performance estimates of abnormal flag for diagnosis of hematological malignancy.

Parameter	Estimate	95% CI
Sensitivity	0.91	0.87–0.94
Specificity	0.89	0.84–0.92
PLR	8.11	5.68–11.58
NLR	0.10	0.07–0.15
DOR	80.28	45.90–140.42

### Clinical utility of abnormal flags for diagnosing hematological malignancy

**Fig 5:**
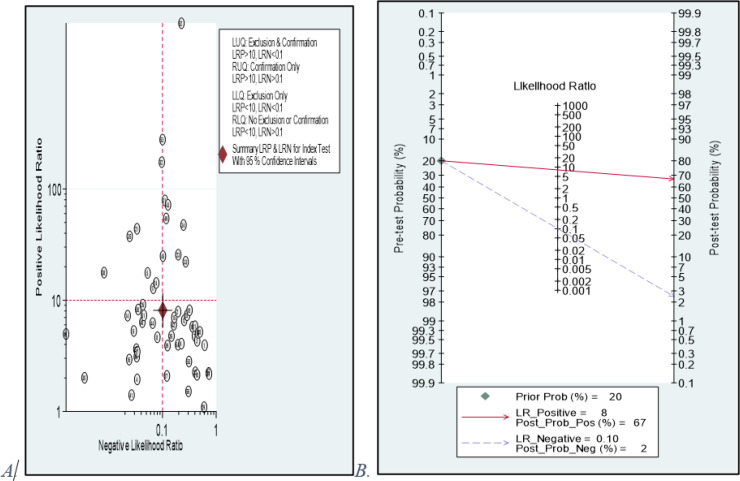
Assessment of the clinical applicability of abnormal flag for the diagnosis of hematological malignancy. (A) Likelihood ratio scattergram. (B) Fagan’s nomogram.

Abnormal hematology analyzer flags serve as important tools in supporting clinical decision-making and improving the diagnostic workflow. To assess their clinical usefulness in detecting hematological malignancies, a likelihood ratio scattergram incorporating both PLR and NLR values was generated. Diagnostic performance is generally regarded as strong when PLR > 10 and NLR < 0.1. In this analysis, six studies—Briggs et al., Schuff-Weiner et al., Schillinger et al., Schapkaitz et al., Joshi et al. (blast flag), and Joshi et al. (abnormal lymphocyte flag) demonstrated particularly high diagnostic accuracy of abnormal flags (Fig 5A).

A Fagan nomogram was also used to further evaluate clinical utility. Assuming a pretest probability of 20%, a positive abnormal flag increased the posttest probability to 67% (PLR = 8), indicating strong rule-in capability. In contrast, a negative flag lowered the posttest probability to 2% (NLR = 0.10), demonstrating excellent rule-out performance. Collectively, these results show that abnormal flagging provides meaningful diagnostic insight and can significantly enhance clinical decision-making when assessing patients for hematological malignancies (Fig 5B).

### Sub group analysis

Studies evaluating the diagnostic accuracy of hematology analyzers demonstrated marked heterogeneity (Q = 1194.284, I^2^ = 100, P < 0.01). To explore possible sources of this variation, subgroup analyses were performed according to continent, publication year, reference standard, analyzer type, and type of abnormal flag assessed.

Subgroup analysis by continent showed that abnormal flags had the highest diagnostic performance in African populations, yielding pooled sensitivity, specificity, and AUC values of 97% (95% CI: 86%–100%), 90% (95% CI: 84%–94%), and 0.96 (95% CI: 0.94–0.98), respectively. In contrast, performance among Asian populations was comparatively lower, with pooled sensitivity and specificity of 88% (95% CI: 81%–92%) and 90% (95% CI: 85%–93%), respectively.

When stratified by reference test, studies using flow cytometry reported the highest sensitivity (99%, 95% CI: 92%–100%), whereas those using microscopic examination showed superior specificity (90%, 95% CI: 86%–94%) and AUC (0.96, 95% CI: 0.94–0.98).

Regarding analyzer type, the Mindray hematology analyzer demonstrated the strongest diagnostic accuracy, with pooled sensitivity, specificity, and AUC of 97% (95% CI: 66%–100%), 98% (95% CI: 96%–99%), and 0.99 (95% CI: 0.98–1.00), respectively. Conversely, the Yumizen H2500 exhibited lower diagnostic performance, with sensitivity 85% (95% CI: 79%–89%), specificity 63% (95% CI: 48%–76%), and AUC 0.86 (95% CI: 0.82–0.88).

Subgroup analysis based on flag type indicated that combined flag parameters offered slightly higher sensitivity (93%, 95% CI: 86%–97%), while immature granulocyte flags produced marginally higher specificity (97%, 95% CI: 88%–99%). Notably, atypical lymphocyte flags showed excellent overall diagnostic performance, with an AUC of 0.98 (95% CI: 0.97–0.99) (Table 3).

**Table 3:** Subgroup analysis of abnormal flagging.

Sub group		No. of studies	Pooled sensitivity (95%CI)	Pooled specificity (95%CI)	PLR (95% CI)	NLR (95% CI)	DOR (95% CI)	AUC (95% CI)	(I^2^)	P-value
Continent	Africa	8	0.97 (0.86, 1.00)	0.90 (0.84, 0.94)	9.8 (6.1,15.6)	0.03 (0.00, 0.16)	348 (69, 1744)	0.96 (0.94, 0.98)	97%	0.000
	Asia	26	0.88 (0.81, 0.92)	0.9 (0.85, 0.93)	8.6 (5.9, 12.4)	0.13 (0.09, 0.21)	63 (33, 122)	0.95 (0.93, 0.96)	99%	0.000
	Europe	20	0.89 (0.80, 0.94)	0.89 (0.75, 0.96)	8.1 (3.4, 19.4)	0.12 (0.07, 0.23)	65 (21, 2061)	0.95 (0.92- 0.96)	100%	0.000
Publication year	≤ 2015	16	0.86 (0.75, 0.93)	0.84 (0.79, 0.88)	5.3 (4.1, 6.9)	0.16 (0.09, 0.30)	33 (17, 64)	0.91 (0.88, 0.93)	99%	0.000
	2016-2020	25	0.92 (0.83, 0.96)	0.89 (0.82, 0.94)	8.6 (4.9- 14.8)	0.09 (0.04, 0.20)	92 (36, 240)	0.96 (0.94, 0.97)	100%	0.000
	2021-2023	16	0.95 (0.86, 0.98)	0.91 (0.84, 0.95)	9.1 (5.4, 16.3)	0.11 (0.02, 0.24)	98 (45, 234)	0.97 (0.95, 0.99)	98%	0.000
Reference tests	Microscope	44	0.92 (0.87, 0.95)	0.90 (0.86, 0.94)	9.6 (6.5, 14.3)	0.09 (0.06, 0.15)	105 (57, 194)	0.96 (0.94, 0.98)	100%	0.000
	Flowcytometer	4	0.99 (0.92, 1.00)	0.71 (0.28, 0.94)	3.5 (0.9, 13.5)	0.01 (0.00, 5.15)	237 (0, 1467)	0.94 (0.91, 0.96)	99%	0.000
	Combined	9	0.83 (0.75, 0.89)	0.84 (0.69, 0.92)	5.1 (2.5 (10.4)	0.20 (0.13, 0.30)	25 (10, 64)	0.89 (0.86, 0.92)	98%	0.000
Hematology analyzer types	Sysmex	37	0.93 (0.87, 0.96)	0.88 (0.82, 0.92)	7.9 (5.1, 12.0)	0.08 (0.04, 0.14)	98 (48,200)	0.96 (0.94, 0.97)	100%	0.000
	Beckman coulter	6	0.86 (0.72, 0.94)	0.92 (0.80, 0.97)	10.6 (4.0, 28.1)	0.15 (0.07, 0.32)	69 (19, 257)	0.95 (0.92, 0.96)	95%	0.000
	Mindary	4	0.97 (0.66, 1.00)	0.98 (0.96, 0.99)	46.7 (23.6, 92.5)	0.03 (0.00, 0.46)	1777 (133, 23799)	0.99 (0.98, 1.00)	93%	0.000
	Advia	4	0.86 (0.60, 0.96)	0.86 (0.53, 0.97)	6.2 (1.5, 25.8)	0.16 (0.05, 0.54)	38 (5, 278)	0.92 (0.90, 0.94)	98%	0.000
	Yumizen H2500	4	0.85 (0.79, 0.89)	0.63 (0.48, 0.76)	2.3 (1.6, 3.4)	0.24 (0.17, 0.33)	10 (5, 18)	0.86 (0.82, 0.88)	90%	0.000
Types of flags	Blast	25	0.89 (0.80, 0.94)	0.90 (0.83, 0.94)	8.9 (5.3, 15.1)	0.12 (0.07, 0.23)	72 (29, 178)	0.95 (0.93, 0.97)	99%	0.000
	Atypical lymphocyte	7	0.93 (0.76, 0.98)	0.97 (0.86, 0.99)	29.2 (6.2, 137.1)	0.07 (0.02, 0.27)	404 (50, 3292)	0.98 (0.97, 0.99)	94%	0.000
	Immature granulocyte	4	0.89 (0.84, 0.93)	0.97 (0.88, 0.99)	27.5 (7.0 (108.1)	0.11 (0.07, 0.17)	252 (48, 1329)	0.94 (0.91, 0.95)	90%	0.000
	Combined	20	93% (86%, 97%)	78% (69%, 85%)	4.2 (3.0, 5.9)	0.09 (0.04, 0.18)	48 (24, 95)	0.92 (0.89, 0.94)	100	0.000

### Meta-regression

**Fig 6:**
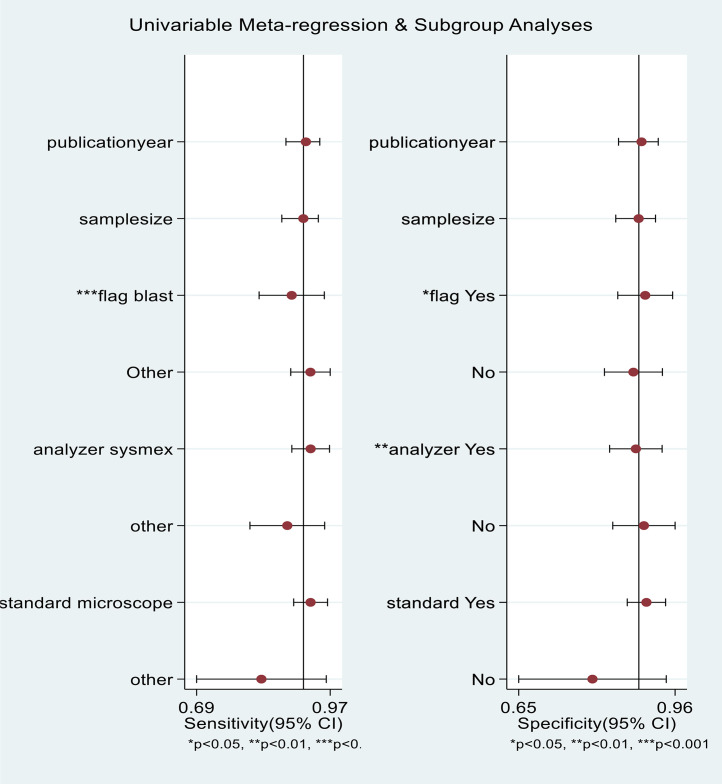
Meta-regression analysis for sensitivity and specificity.

The type of abnormal flag was identified as a significant contributor to heterogeneity in sensitivity (p < 0.001). Moreover, both the type of abnormal flag (p < 0.05) and the type of hematology analyzer (p < 0.01) were found to significantly influence specificity (Fig 6).

### Sensitivity analysis

**Fig 7:**
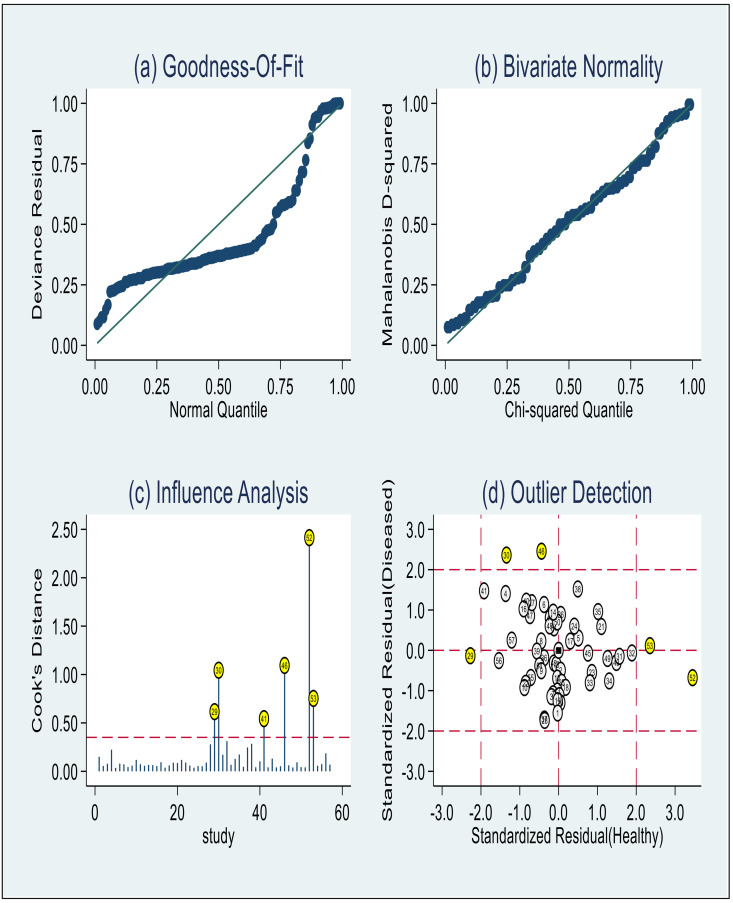
Sensitivity analysis of abnormal flagging for hematological malignancy.

The results of the sensitivity analysis are presented in Fig 7. Assessment of model fit (Fig 7A) and evaluation of bivariate normality (Fig 7B) confirmed the robustness of the selected model. Influence analysis indicated that the studies by Eilertsen et al. (2019), Petrone et al. (2019), Bloome et al. (2020), Ramiah et al. (2023), Schoorl et al. (2025a), and Schoorl et al. (2025b) had the greatest impact on the overall results (Fig 7C). Outlier detection suggested that the observed heterogeneity could be primarily attributed to the data from Eilertsen et al. (2019), Petrone et al. (2019), Ramiah et al. (2023), Schoorl et al. (2025a), and Schoorl et al. (2025b) (Fig 7D). Exclusion of these five outlier studies yielded pooled estimates of sensitivity (0.90, 95% CI: 0.85–0.94), specificity (0.88, 95% CI: 0.84–0.91), PLR (7.6, 95% CI: 5.6–10.2), NLR (0.11, 95% CI: 0.07–0.17), DOR (68, 95% CI: 40–115), and AUC (0.95, 95% CI: 0.93–0.97). Importantly, the exclusion of these outliers did not result in any substantial changes to the overall pooled diagnostic performance, confirming the stability and reliability of the meta-analysis results (Fig 7).

### Publication bias

**Fig 8:**
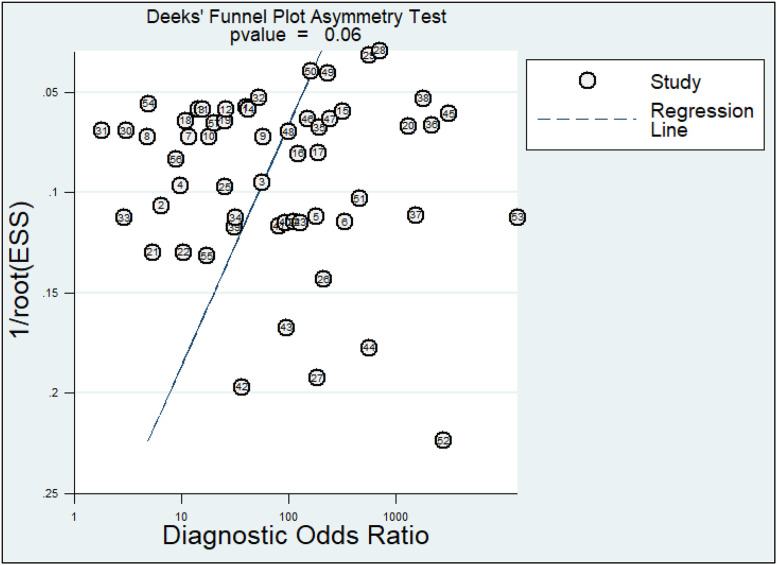
Deeks’ funnel plot for publication bias.

Deeks’ funnel plot was constructed to assess the potential for publication bias in this study. The included studies showed a relatively symmetric distribution around the regression line, with a p-value of 0.06. These differences were not statistically significant, indicating that there was no evidence of publication bias in the included study (Fig 8).

## Discussion

This systematic review and meta-analysis evaluated the diagnostic accuracy of automated hematology analyzer abnormal flags for detecting hematological malignancies. Overall, pooled estimates indicated high sensitivity (91%) and specificity (89%), with an AUC of 0.96, suggesting that abnormal flags may have value as screening indicators for identifying samples requiring further hematological evaluation. Nevertheless, interpretation of these pooled estimates requires caution because substantial heterogeneity was observed across included studies. Variations in analyzer technologies, flagging algorithms, disease spectra, patient populations, and reference standards likely contributed to the observed variability and may limit the generalizability of the pooled results. These results are consistent with earlier research that showed the diagnostic accuracy of abnormal flags produced by analyzers. Advanced algorithms and multi-angle light scatter technology are used by contemporary hematology analyzers, like the Sysmex XN-series and Beckman Coulter DxH, to identify atypical cells, blasts, and other aberrant populations [47,48].

An important consideration is the clinical heterogeneity among the included studies. The meta-analysis combined investigations evaluating different hematological malignancies, including acute and chronic leukemias, myelodysplastic syndromes, chronic myelomonocytic leukemia, and other hematologic disorders. Likewise, studies assessed diverse abnormal flag categories, including blast flags, abnormal lymphocyte flags, immature granulocyte flags, monocytosis-related flags, and combinations of multiple flag parameters. Because these flags are designed to detect distinct cellular abnormalities and may perform differently across disease categories, the pooled estimates should be regarded as an overall measure of screening performance rather than disease-specific diagnostic accuracy. The observed diagnostic performance suggests that abnormal flags can contribute meaningfully to early laboratory recognition of potentially malignant hematologic conditions. However, abnormal flagging should be viewed primarily as a screening rather than a standalone diagnostic approach. Positive flags require confirmation through established diagnostic procedures, including peripheral blood smear review, flow cytometry, immunophenotyping, bone marrow examination, and molecular testing, depending on the clinical context.

The pooled AUC of 0.96 indicates that abnormal flags are approaching the diagnostic precision of more advanced assays, particularly when it comes to identifying cases requiring rapid investigation. Reactive disorders, such as infections or inflammation, might give false-positive results, whereas early-stage or low-tumor-burden diseases can produce false-negative outcomes.

The studies exhibited significant heterogeneity (I^2^ = 94.48% for sensitivity and 99.73% for specificity). This could be attributed to variances in sample populations, flagging algorithms, and analyzer models. Variability may also be raised by employing different diagnostic cutoffs and include both adult and child cohorts. Additionally, the included studies used heterogeneous reference standards, ranging from peripheral blood smear review to flow cytometry, bone marrow examination, and immunophenotyping. Such variation may have influenced reported diagnostic accuracy and contributed to heterogeneity. A bivariate random-effects model was employed to address issue, producing credible pooled estimates.

Diagnostic performance varied significantly between continents, according to subgroup analyses. With an AUC of 0.96 and pooled sensitivity and specificity of 97% and 90%, abnormal flags demonstrated the highest diagnosis accuracy among African patients. This improved performance could be attributed to changes in disease burden, population characteristics, or analyzer calibration settings unique to African laboratories. In contrast, studies conducted in Asia demonstrated slightly reduced diagnosis accuracy, indicating potential changes in case mix, disease prevalence, or reference standard application.

The reference test utilized to confirm hematological malignancy had a significant impact on the diagnostic outcomes. When flow cytometry was utilized as the reference method, abnormal flags revealed a remarkable high sensitivity (99%), showing their efficacy as a preliminary screening tool for identifying potential malignancies that require further confirming testing. However, when microscopic examination was used as the reference test, the specificity and AUC were greater (90% and 0.96, respectively), suggesting the excellent accuracy of manual morphological assessment in ruling out false positives. The included studies employed a variety of reference standards, including peripheral blood smear review, flow cytometry, bone marrow examination, immunophenotyping, and combined diagnostic approaches. Because these methods differ in their ability to detect hematological malignancies, variation in reference standards may have contributed to heterogeneity and influenced pooled diagnostic accuracy estimates. Future studies would benefit from greater standardization of reference diagnostic criteria.

The type of analyzer utilized has an impact on diagnosis accuracy as well. The Mindray analyzer fared better, with 97% sensitivity, 98% specificity, and 0.99 AUC, respectively. These findings demonstrate that newer-generation analyzers with improved algorithms and abnormal cell flagging capabilities have advanced. In contrast, the Yumizen H2500 analyzer fared worse, which could be due to changes in detection algorithms, sample throughput, or flagging levels. These disparities underscore the importance of analyzer selection and calibration in maximizing diagnostic outcomes.

In terms of flag type, the combination of flag parameters resulted in a slightly better sensitivity (93%), implying that detecting hematological malignancies is improved by integrating numerous aberrant cell flags. Immature granulocyte flags had higher specificity (97%), indicating that they can detect reactive hematological changes with fewer false positives. Significantly, the overall diagnostic accuracy of atypical lymphocyte flags was outstanding (AUC = 0.98), showing that they are especially beneficial in diagnosing lymphoid malignancies, which are usually associated with unusual patterns. These findings show that abnormal flagging on automated hematology analyzers is a reliable and effective first screening tool for hematological malignancies, particularly when utilized with appropriate confirmatory procedures such as peripheral smear review or flow cytometry. However, the observed variation emphasizes the necessity for standardization of flagging algorithms, device calibration, and diagnostic criteria across different laboratories and geographies.

Subgroup analyses suggested variability in diagnostic performance according to analyzer platform, reference standard, and flag type. However, these findings should be interpreted cautiously because several subgroups included relatively few studies and remained highly heterogeneous. Consequently, observed differences may reflect methodological variation and study-specific characteristics rather than true differences in diagnostic performance.

A meta-regression analysis was conducted to identify potential sources of heterogeneity among the included papers. The type of aberrant flag significantly influenced the heterogeneity in sensitivity (p < 0.001). This finding suggests that modifications in flagging algorithms or the specific parameters utilized to detect aberrant cells may impact the analyzers’ sensitivity for detecting hematological cancers. Higher sensitivity across studies can be achieved by detecting early or subtle malignant morphological alterations using certain flag types, such as those that recognize abnormal lymphocytes or blasts. The type of aberrant flag (p < 0.05) and hematology analyzer type (p < 0.01) both had a significant impact on specificity. This shows that differences in analyzer technology, signal detecting techniques, and algorithm sophistication contribute to diagnostic precision variability. To reduce false-positive rates, analyzers with more complex detection systems and enhanced flagging criteria may be able to differentiate between reactive and malignant hematological alterations. These findings show that the design and functioning of the analyzer have an impact on.

Sensitivity analysis provided additional support for the pooled estimates’ robustness. Several studies, including Eilertsen et al. (2019), Petrone et al. (2019), Ramiah et al. (2023), and Schoorl et al. (2025a, 2025b), were discovered to have the greatest impact on heterogeneity through influence and outlier detection. Excluding these studies had no significant effect on the pooled sensitivity, specificity, PLR, NLR, DOR, and AUC, indicating that the overall findings are consistent and credible.

Abnormal flags’ clinical value was further demonstrated using likelihood ratio scattergrams and Fagan nomogram analyses. A positive abnormal flag increased the posttest probability to 67% (PLR = 8) from a pretest probability of 20%, suggesting good rule in capacity, whereas a negative flag reduced the posttest probability to 2% (NLR = 0.10), indicating excellent rule out performance. These findings highlight the utility of aberrant flags in guiding medical decision-making. Clinical utility analyses indicated that abnormal flags may contribute to both ruling in and ruling out hematological malignancies when incorporated into routine laboratory workflows. Nevertheless, these findings should be interpreted as supportive evidence rather than proof of independent diagnostic capability. The utility of abnormal flags is likely greatest when integrated with clinical assessment, peripheral blood smear review, and confirmatory laboratory investigations.

Finally, there was no evidence of publication bias in Deeks’ funnel plot study. The non-significant p-value (0.06) suggests that selective reporting had no effect on the results. The included studies were symmetrically distributed around the regression line.

Overall, abnormal flagging on hematology analyzers is a dependable and practical clinical technique for early detection of hematological tumors. Flag type, analyzer technology, and reference test all have an impact on diagnostic performance, emphasizing the importance of standardizing calibration, harmonizing flagging algorithms, and undertaking additional multicenter validation studies to maximize clinical utility.

This review has several strengths, including comprehensive literature searching, application of QUADAS-2 methodology, use of a bivariate random-effects diagnostic meta-analysis model, subgroup analyses, meta-regression, sensitivity analyses, and evaluation of clinical utility. However, several limitations should be acknowledged. First, substantial statistical heterogeneity was present across studies. Second, clinically heterogeneous diseases and abnormal flag types were pooled. Third, reference standards were not uniform across investigations. Fourth, some studies contributed multiple datasets derived from the same patient population, potentially affecting the assumption of independence. Finally, several subgroup analyses included relatively small numbers of studies. These limitations may influence the precision and generalizability of the pooled estimates. Despite these limitations, the findings provide solid evidence of the clinical utility and diagnostic accuracy of abnormal hematology analyzer flags in detecting hematological malignancies.

## Conclusion and recommendation

This meta-analysis demonstrates that abnormal flagging in hematology analyzers is a reliable and clinically helpful strategy for early diagnosis of hematological cancers. Abnormal flags exhibit high sensitivity, specificity, and overall diagnostic accuracy, particularly when related with sophisticated analyzers like Mindray and certain flag types, such as abnormal lymphocytes or blasts. The clinical utility is further reinforced by likelihood ratio and Fagan nomogram studies, which demonstrate their robust rule-in and rule-out capabilities, allowing for more informed clinical decision-making. Incorporating analyzer flagging into routine hematological procedures, especially in situations with limited resources, may promote early detection, maximize laboratory efficiency, and assist rapid clinical decision-making.

The observed variation emphasizes the importance to standardize flagging algorithms, instrument calibration, and diagnostic criteria across laboratories and geographies. More multicenter trials with defined methodologies are needed to validate these findings and improve the clinical value of abnormal flagging as an early detection tool for hematological cancers. As a result, harmonizing analyzer calibration, optimizing flagging algorithms, and testing anomalous flag criteria across populations are critical steps toward improving the reliability and clinical utility of hematology analyzers in the diagnosis of hematological malignancies.

Generally, our findings demonstrate that automated hematology analyzer abnormal flags demonstrated promising diagnostic performance; however, substantial heterogeneity limits the certainty and generalizability of pooled estimates. Abnormal flags should be viewed as supportive screening indicators that assist laboratory triage and prioritize further investigation. Definitive diagnosis of hematological malignancies continues to require confirmatory methods such as peripheral blood smear examination, flow cytometry, immunophenotyping, molecular testing, and bone marrow evaluation where clinically indicated. To maximize the diagnostic value of abnormal flags in early detection and diagnosis of hematological malignancies, laboratory standardization and additional multicenter validation studies are necessary.

## Supporting information

S1 File PRISMA Checklist For “Diagnostic Accuracy of Automated Hematology Analyzer Abnormal Flags for Detecting Hematological Malignancies: A Systematic Review and Meta-Analysis”. (Supporting information 1.DOCX)

S2 File Search strategy used to retrieve eligible studies for Diagnostic Accuracy of Automated Hematology Analyzer Abnormal Flags for Detecting Hematological Malignancies: A Systematic Review and Meta-Analysis. (Supporting information 2.DOCX)
