# Supplementary material for: Diagnostic accuracy of automated hematology analyzer abnormal flags for detecting hematological malignancies: A systematic review and meta-analysis
Source: PLoS One. 2026 Jul 31;21(7):e0354619. doi: 10.1371/journal.pone.0354619 (PMC13426980; doi:10.1371/journal.pone.0354619)
Supplement: S2 File — (Supporting information 2.DOCX) [file pone.0354619.s002.docx]

**Supporting information 2: Search strategy used to retrieve eligible studies for Diagnostic Accuracy of Automated Hematology Analyzer Abnormal Flags for Detecting Hematological Malignancies: A Systematic Review and Meta-Analysis.**

| **Electronic database** | **Combination** | **Number of article** | **Last searching date** |
| --- | --- | --- | --- |
| PubMed | ((((((((((((("hematology analyzer"[Title/Abstract] OR "haematology analyzer"[Title/Abstract] OR "abnormal flag*"[Title/Abstract] OR "blast flag*"[Title/Abstract] OR "WBC flag*"[Title/Abstract] OR "atypical lymphocyte*"[Title/Abstract] OR "abnormal lymphocyte*"[Title/Abstract] OR monocytosis[Title/Abstract]) AND (leukemia[Title/Abstract] OR leukaemia[Title/Abstract] OR lymphoma[Title/Abstract]  OR "hematological malignancy*"[Title/Abstract] OR "haematological malignancy*"[Title/Abstract] OR "myelodysplastic syndrome*"[Title/Abstract]  OR myelodysplasia[Title/Abstract]) AND (Humans[Mesh]) AND (English[Filter]) NOT (preprint[Publication Type])))) | 982 | October 30th, 2025 |
| EMBASE | ((Abstract:("hematological analyzer")) OR (flag) OR ("blast flag") OR (WPC) OR ("atypical lymphocyte") OR ("abnormal lymphocyte")) AND (("hematological malignancy") OR (white blood cell disorders') OR (leukemia) OR (lymphoma) OR (myelodysplasia)) AND (("diagnostic accuracy") OR (performance)) | 104 | October 30th, 2025 |
| Cochrane | "haematology analyzer" OR flag* OR "abnormal flagging" OR "blast flag" OR "WBC flag" OR "alarm flag" OR WPC) AND ("hematologic malignancy" OR white blood cell disorders' OR "leukemia" OR "lymphoma" OR "myelodysplasia") AND (sensitivity OR specificity OR "diagnostic accuracy" OR "predictive value" OR performance) | 65 |  |
| Scopus | (TITLE-ABS-KEY (“haematology analyzer") OR TITLE-ABS-KEY ("flag") OR TITLE-ABS-KEY ("abnormal flagging") OR TITLE-ABS-KEY ("blast flag") OR TITLE-ABS-KEY ("alarm flag") OR TITLE-ABS-KEY ("WPC") AND TITLE-ABS-KEY ("hematologic malignancy") TITLE-ABS-KEY ("white blood cell disorders") OR TITLE-ABS-KEY ("leukemia") OR TITLE-ABS-KEY ("lymphoma") OR TITLE-ABS-KEY ("myelodysplasia") AND TITLE-ABS-KEY ("sensitivity") OR TITLE-ABS-KEY ("specificity") OR TITLE-ABS-KEY ("diagnostic accuracy") OR TITLE-ABS-KEY ("predictive value") OR TITLE-ABS-KEY ("("predictive value"))) | 115 | October 30th, 2025 |
| Other sources | " haematology analyzer"; flag; abnormal flagging; "blast flag"; "alarm flag"; WPC; "hematologic malignancy"; "white blood cell disorders"; “multiple myeloma”; myeloproliferative; leukemia; lymphoma; myelodysplasia; sensitivity; specificity; "diagnostic accuracy" and "predictive value") | 21 | October 30th, 2025 |
