# Supplementary material for: Diagnostic accuracy of automated hematology analyzer abnormal flags for detecting hematological malignancies: A systematic review and meta-analysis
Source: PLoS One. 2026 Jul 31;21(7):e0354619. doi: 10.1371/journal.pone.0354619 (PMC13426980; doi:10.1371/journal.pone.0354619)
Supplement: Attachment — Submitted filename: Response to Reviewers.docx [file pone.0354619.s004.docx]

**Author’s Response to editor and Reviewers**

PONE-D-26-09272

**Diagnostic Accuracy of Automated Hematology Analyzer Abnormal Flags for Detecting Hematological Malignancies: A Systematic Review and Meta-Analysis**

**Zewudu Mulatie^1*^, Bruktawit Eshetu^1^,** **Afewerk Habtamu Aseme^1^,** **Sisay Desale^1^,** **Saleamlak Sebsibe^1^,** **Yonas Erkihun^1^, Yeshimebet Kassa^1^, Tesfaye Gessese^1^, Mihreteab Alebachew^1^, Mikiyas Shimeles^1^,** **Mahider Shimelis Feyisa^2^, Dereje Mengesha Berta^3^**

**PLOSE ONE**

***Corresponding Author**: Zewudu Mulatie

Department of Medical Laboratory Sciences, College of Medicine and Health Science, Wollo University, Dessie, Ethiopia. P.O.Box 1145; email: [zewudumulatie@gmail.com](mailto:zewudumulatie@gmail.com) ; Tel: +251945274251

**Dear PLOSE ONE** **Journal Editorial Office**

We would like to express our gratitude to the editorial teams and reviewers for their insightful comments and suggestions, which helped to make our manuscript, " **Diagnostic Accuracy of Automated Hematology Analyzer Abnormal Flags for Detecting Hematological Malignancies: A Systematic Review and Meta-Analysis**," more valuable and scientifically sound. The authors, tried to correct and include all the comments given by the reviewer point-by-point. For convenience purposes, we attached or uploaded the reply to the reviewer and the revised manuscript with track changes. We confirm that this manuscript has not been published elsewhere and is not under consideration by another journal. All authors have approved the manuscript and agreed with its resubmission to the PLOSE ONE Journal. All the reviewer comment responses are presented below.

**With regards!**

Zewudu Mulatie

Corresponding author

**Response to editor’s**

We thank you very much for your constructive comments and suggestions for the improvement of this manuscript. We have corrected and amended the manuscript based on your comments and suggestions. Herewith, please find in blue, the point-by-point response for editor’s comments and suggestions.

**Editor comments**

**Comment 1:** 1. Please ensure that your manuscript meets PLOS ONE's style requirements, including those for file naming. The PLOS ONE style templates can be found at

<https://journals.plos.org/plosone/s/file?id=wjVg/PLOSOne_formatting_sample_main_body.pdf> and

[https://journals.plos.org/plosone/s/file?id=ba62/PLOSOne_formatting_sample_title_authors_affiliations.pdf](https://track.editorialmanager.com/CL0/https:%2F%2Fjournals.plos.org%2Fplosone%2Fs%2Ffile%3Fid=ba62%2FPLOSOne_formatting_sample_title_authors_affiliations.pdf/1/010f019eb61bc7ca-7a149499-8349-47dd-93ee-428566255b14-000000/MSFQcDBMp45z0w650J_OwgYeXUE9Nc-FJEfTEF8rN9k=258).

***Response****:* We would like to thank you for your suggestion. We tried address your suggestion in the manuscript*.*

2. We note that your Data Availability Statement is currently as follows: [All relevant data are within the manuscript and its Supporting Information files.]

Please confirm at this time whether or not your submission contains all raw data required to replicate the results of your study. Authors must share the “minimal data set” for their submission. PLOS defines the minimal data set to consist of the data required to replicate all study findings reported in the article, as well as related metadata and methods (<https://journals.plos.org/plosone/s/data-availability#loc-minimal-data-set-definition>).

***Response****:* We would like to thank you for your suggestion. We tried to upload the minimal data 3. Please include captions for your Supporting Information files at the end of your manuscript, and update any in-text citations to match accordingly. Please see our Supporting Information guidelines for more information: [http://journals.plos.org/plosone/s/supporting-information](http://track.editorialmanager.com/CL0/http:%2F%2Fjournals.plos.org%2Fplosone%2Fs%2Fsupporting-information/1/010f019eb61bc7ca-7a149499-8349-47dd-93ee-428566255b14-000000/FaA32RbPVZ9-sgko9tHC6c4cLL5376_7NANulJ399xA=258).set in to the system*.*

***Response****:* We would like to thank you for your suggestion. We tried address your suggestion in the manuscript*.*

**Response to reviewer(s’) comments**

On behave of the authors, I would like to thank reviewers for their constructive and scholarly comments that are valuable to make the paper scientifically sound. The comments are well accepted and most of the suggestions made by the reviewers were incorporated. Those changes are highlighted in blue within the manuscript. Herewith, please find in blue, the point-by-point response for reviewers’ comments and suggestions.

**REVIEWER #1 General comments**

We sincerely thank the reviewer for the thorough and constructive evaluation of our manuscript. We greatly appreciate the positive comments regarding the clinical relevance of our study and its potential value for laboratories, particularly in resource-limited settings. We also thank the reviewer for recognizing the strengths of our work, including the application of standard diagnostic test accuracy meta-analysis methods, subgroup analyses, and clinical utility assessments.

We have carefully considered all of the reviewer's comments and have substantially revised the manuscript to improve its methodological rigor, transparency, and overall presentation. Specifically:

**Comment 1: “**extremely high heterogeneity.”

***Response****:* We would like to thank you for your suggestion. We provide a more comprehensive discussion of the potential clinical and methodological sources of heterogeneity and explicitly acknowledge its implications for interpretation of the pooled estimates.

***Comment 2:*** pooling of clinically heterogeneous conditions and flag types,

***Response****:* We would like to thank you for your suggestion. We have clarified the rationale for data pooling and performed additional subgroup analyses according to analyzer flag category, and analyzer platform where sufficient studies were available. The limitations associated with combining heterogeneous disease entities and flag types are now more clearly discussed.

***Comment 3:*** *inconsistent reference standards,*

***Response****:* We would like to thank the reviewer for the comments. We discussed the variability in reference standards as a potential contributor to heterogeneity and its possible influence on diagnostic accuracy estimates.

***Comment 4:*** *possible non-independence of datasets,*

***Response****:* We would like to thank the reviewer for your suggestion. We carefully re-examined all included studies to identify multiple datasets originating from the same publication. These datasets are now clearly identified in the study characteristics table, and we have clarified in the methods how they were handled to minimize potential bias arising from non-independence.

***Comment 5:***	*insufficient methodological transparency.*

**Response**: We would like to thank the reviewer for the comments. We have substantially expanded the Methods section to provide additional details regarding literature searching, study selection, data extraction, quality assessment, statistical analyses, subgroup analyses, meta-regression, sensitivity analyses, publication bias assessment, and clinical utility evaluation. Reporting throughout the manuscript has been revised to improve transparency and reproducibility.

***Comment 6:*** *overinterpretation of pooled findings.*

**Response**: We would like to thank the reviewer for the comments. We have revised the results and discussion to present a more balanced interpretation of the pooled diagnostic performance. The conclusions have been moderated to better reflect the observed heterogeneity and limitations of the available evidence, avoiding overstatement of the findings.

***Comment 7:*** *In addition, the manuscript requires substantial language editing and restructuring of the discussion section to reduce repetition and improve analytical depth.*

**Response**: We would like to thank the reviewer for the suggestion. The manuscript has undergone comprehensive language editing to improve grammar, clarity, and readability. In addition, the discussion has been substantially reorganized to reduce repetition, improve logical flow, provide deeper interpretation of the findings, and better integrate comparisons with previous studies while emphasizing the strengths and limitations of the evidence.

**REVIEWER #2 General comments and suggestions**

We sincerely thank the reviewer for the thorough and constructive evaluation of our manuscript. We greatly appreciate the positive comments regarding the clinical relevance of our study and its potential value for laboratories, particularly in resource-limited settings.

We have carefully considered all of the reviewer's comments and have substantially revised the manuscript to improve for publication. Specifically:

***Comment*** 1: The search strategy requires clarification. The supplementary search strategy includes a randomized controlled trial filter in PubMed despite the review focusing on diagnostic accuracy studies. The rationale for this approach should be explained, and the authors should discuss whether relevant studies may have been inadvertently excluded. In addition, the search syntax contains several formatting inconsistencies and should be presented in a fully reproducible manner.

***Response****:* We would like to thank you for identifying this issue. Upon re-examination of the search strategy, we confirmed that the randomized controlled trial (RCT) filter was inadvertently retained from a previously developed search template and was not appropriate for a diagnostic test accuracy review. The search strategy has therefore been revised and simplified to focus exclusively on diagnostic accuracy studies involving hematology analyzer abnormal flags and hematological malignancies.

To ensure transparency and reproducibility, we have provided the complete database-specific search strategies in a supplementary file, including all search terms, Boolean operators, truncations, and field tags. We also re-ran the search using the revised strategy and verified that no eligible studies were missed.

***Comment*** 2: The pooled analyses demonstrate extremely high heterogeneity for both sensitivity and specificity. Although subgroup analyses and meta-regression were performed, the manuscript would benefit from a more detailed discussion of the sources of heterogeneity and their implications for interpretation of the pooled estimates.

***Response****:* We would like to thank you for your comments. We have substantially expanded the discussion section to provide a more comprehensive examination of heterogeneity. Potential sources of heterogeneity include differences in analyzer platforms, flagging algorithms, disease categories, patient populations, study designs, and reference standards. We have also emphasized that the pooled estimates should be interpreted cautiously because of the substantial between-study variability.
***Comment*** 3: Several publications contributed multiple datasets to the meta-analysis. The authors should clarify how these datasets were handled statistically and discuss any potential impact on the pooled results.

***Response****:* We would like to thank you for your comments. We appreciate this important observation. Several studies reported diagnostic performance for multiple analyzer platforms or abnormal flag categories using the same study population. These datasets were treated as separate index-test evaluations because they represented distinct diagnostic tests. However, we acknowledge that this approach may introduce partial non-independence and potentially increase the influence of certain study populations on pooled estimates.

***Comment*** 4: The included studies used different reference standards, including microscopy, flow cytometry, bone marrow examination, and combined approaches. The potential influence of this variability on diagnostic accuracy estimates should be discussed more thoroughly.

***Response****:* We would like to thank you for your suggestion. We agree with the reviewer. The variability in reference standards represents an important source of heterogeneity and may influence diagnostic accuracy estimates. We have expanded the Discussion section to explain how differences in reference standards could affect sensitivity and specificity and potentially contribute to variability across studies.

***Comment*** 5: The conclusions should be moderated to emphasize that abnormal analyzer flags are useful screening tools but cannot replace confirmatory diagnostic methods such as peripheral blood smear review, flow cytometry, or bone marrow examination.

***Response****:* We would like to thank you for your suggestion. The abstract, discussion, and conclusion sections have been revised to avoid overinterpretation and to emphasize that abnormal flags should be considered screening or triage tools rather than definitive diagnostic tests.
**Minor Comment**

***Comment*** 1: The manuscript requires substantial English-language editing.

***Response****:* We would like to thank you for your comments. We tried to edit the manuscript.
***Comment*** 2: Terminology should be standardized throughout the manuscript.
***Response****:* We would like to thank you for your concern. We tried standardize terminology throughout the manuscript.

***Comment*** 3: Table 1 would benefit from improved formatting and clearer presentation of multiple datasets originating from the same publication.

***Response****:* We would like to thank you for your comments. Table 1 has been reformatted to improve readability and to clearly indicate multiple datasets derived from the same publication.
